# Low Efficacy of Periodical Thermal Shock for Long-Term Control of *Legionella* spp. in Hot Water System of Hotels

**DOI:** 10.3390/pathogens11020152

**Published:** 2022-01-25

**Authors:** Jhon J. Molina, Magdalena Bennassar, Edwin Palacio, Sebastian Crespi

**Affiliations:** 1 Biolinea Int., 07007 Palma, Spain; j.molina@uib.es (J.J.M.); laboratorio@biolinea.com (M.B.); 2 Environmental Analytical Chemistry Group, University of the Balearic Islands, 07122 Palma, Spain; edwin.palacio@uib.es

**Keywords:** *Legionella*, thermal shock, hot water system, hotel facilities

## Abstract

Different guidelines and regulations for the prevention of legionellosis in public facilities include the recommendation of a periodical thermal shock in the hot water system. The purpose of this study was to assess the efficacy of periodical thermal shocks along a 1-year period on the presence of *Legionella* spp. in the domestic hot water system of hotels. The Legionella testing results from the period January–December 2019 coming from a group of 77 hotel facilities in the Balearic Islands (Spain) conducting periodical thermal shocks were analyzed. A second group of 44 hotels operating without periodical thermal shocks was used for a comparative analysis. In the facilities where the periodical thermal shock was performed, 16.0% of the results (429 hot water samples collected) were positive for *Legionella* spp., compared to 21.1% (298 samples), where periodical thermal shock was not performed. Overall, in the thermal shock group, 32.5% of the sites presented at least 1 positive sample along the period of study versus 45.5% in the control group. None of these differences was statistically significant (*p*-value > 0.05). These findings suggest that the efficacy of regular thermal shock for long-term control of *Legionella* spp. in domestic hot water systems of hotels is low.

## 1. Introduction

Legionellae are Gram-negative bacteria commonly found in natural aquatic environments, including the usual drinking-water catchment areas, such as lakes or rivers. From there, it can colonize the supply distribution systems, and subsequently, the domestic distribution networks, and other engineered water systems that may require water for their operation [1].

In Europe, approximately 20% of the reported cases of legionellosis are associated with travel. Hotels and other commercial accommodation sites (camping sites, holiday resorts, etc.) have often been associated with clusters and outbreaks of Legionnaires’ disease [2]. Various studies have indicated a high prevalence of *Legionella* spp. in domestic distribution systems of hotels, particularly in hot water systems [3,4]. The building size, stagnation, corrosion, water temperatures between 25 and 45 °C and the presence of dirt and nutrients have been associated with the risk of colonization [5,6]. Different strategies to prevent colonization and subsequent growth of *Legionella* in hot water systems have been proposed, chief among them being the maintenance of high temperatures (50–60 °C) [7]. Recently, a new cut-off of 55 °C has been proposed for hotels, similar to that used for many health care settings, given the high probability of obtaining positive *Legionella* results at temperatures below 55 °C [8].

Either to improve the effectiveness of the permanent maintenance of high temperatures (50–60 °C) or to be used as an alternative method of control, e.g., for saving energy purposes, a periodical thermal shock—increasing hot water temperatures at >60 °C for short periods at regular intervals—has been included in different guidelines and regulations as part of long-term control programs for domestic hot water systems [9]. In Spain, a monthly thermal shock at 70 °C for 2 h is recommended by the official technical guidelines in addition to the permanent maintenance of a 50–60 °C range in the hot water network [10,11]. The procedure followed for regular thermal shocks used to be a variant of the full thermal disinfection method commonly used for urgent remedial purposes, but in this case, the end points are not flushed, and the times and temperatures may be lower. In many installations, this procedure is currently programmed into the electronic building management system and is carried out automatically.

However, this methodology has not been adopted universally, and its usefulness has been disputed on both practical and empirical grounds. First, several case studies, carried out mainly in hospitals and lab-scale units, have reported limited, short-term effects in reducing colonization levels [12,13]. Moreover, the *ESGLI European Technical Guidelines for the Prevention, Control and Investigation, of Infections Caused by Legionella species* (ESGLI European Technical Guidelines) recommends against its use as part of a long-term control program based on the occurrence of frequent and rapid recolonizations and the increased risk of scalding carried by the procedure [14]. In addition, different studies on thermal shock procedures, both in vitro and field studies, have reported potentially relevant side effects, such as rapid regrowth with subsequent increase in *Legionella* concentrations [15], selection for thermal tolerance of *L. pneumophila* species [16] and increase in the relative abundance of amoebae [17]. Finally, and most important, there are no comparative field studies supporting its efficacy for the control of *Legionella* in hot water systems. Taken together, all these facts and matters should be a call to caution when considering the inclusion of regular thermal shocks in *Legionella* prevention plans.

In this context of scarce and conflicting evidence, further research is needed to clarify whether thermal shocks add any real benefit or not when included in preventive plans. In the first place, it is necessary to elucidate whether the procedure is effective, considering real field experiences, including different establishments. The purpose of the present study is to evaluate the usefulness of regular thermal shocks in hotels in terms of their ability to eliminate or reduce the colonization of *Legionella* in the hot water systems. To our knowledge, this is the first field study evaluating the impact of regular thermal shocks on the level of colonization by *Legionella* spp. in hotel hot water systems.

## 2. Results

### 2.1. Characterization of the Hotel Groups

A total of 121 hotels were included in the study. Group A included 77 hotels that carried out regular thermal shocks and group B included 44 hotels without regular thermal shock. In group A, 59.7% performed thermal shock monthly, 24.7% weekly, 5.2% biweekly, and 10.4% at other frequencies (daily, quarterly, every other day, etc.). Average hot water temperatures were 55.8 ± 6.1 °C in group A and 55.2 ± 5.6 °C in group B. The proportion of samples collected from each group in the different temperature ranges is shown in Table 1.

### 2.2. Qualitative Results

**Figure 1:**
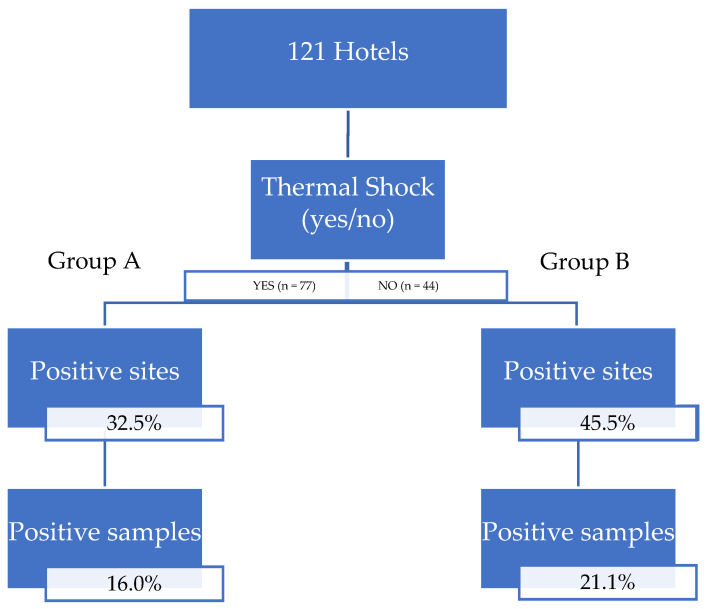
Diagram showing the overall qualitative results in groups A and B.

In total, 429 water samples were collected from group A and 298 from group B. In group A, 16.0% of the samples were positive for *Legionella* spp. and 32.5% of the sites presented at least one sample as being positive. In group B, the figures were 21.1% and 45.5%, respectively (Figure 1). There were no statistically significant differences between the two groups for any of the two analyzed parameters (*p* > 0.05).

Table 2 shows the proportion of positive samples in both groups in relation to the main sampling points: showers, taps, calorifiers and return lines.

Table 3 shows the average temperatures of the positive and negative samples of groups A and B.

### 2.3. Quantitative Results

The quantitative *Legionella* spp. results (CFU/L) for both groups are summarized in Table 4.

**Figure 2:**
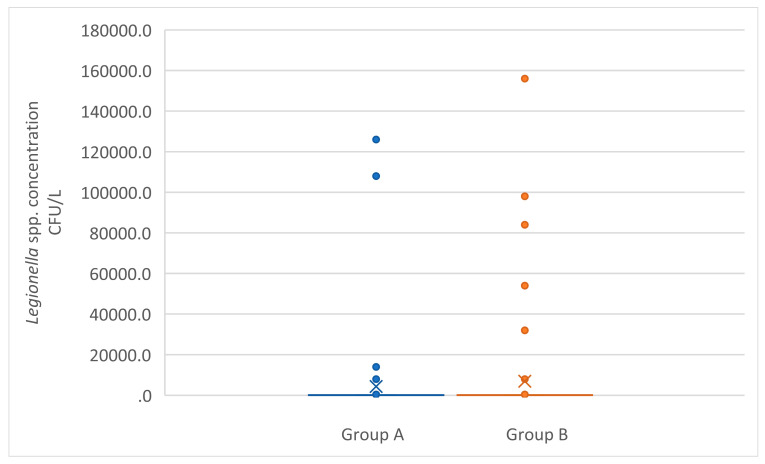
Box plot of *Legionella* spp. concentrations (CFU/L) in positive samples of groups A and B.

When the overall results were included in the comparative analysis, there were no differences between the two groups (*p* > 0.05). When only positive results were considered, the concentrations in group B were higher than the concentrations in group A, showing a statistically significant increase (Mann–Whitney U test, *p* = 0.0094) (Figure 2).

## 3. Discussion

Thermal shock treatment at temperatures ≥ 70 °C for relatively short periods has been used for emergency disinfection and as part of long-term control programs. However, whilst for emergency disinfection, the procedure can be well justified based on the potential imminent risk, due to high colonization or facing outbreak situations, their use for prevention purposes is more debatable, given the absence of studies supporting its efficacy.

Despite this, in recent years, different regulations and guidelines have included periodical thermal shocks as part of the routine maintenance of hot water systems, although both the frequency and the conditions (temperatures and times) vary widely. In Italy, the *Linee Guida per la Prevenzione ed il Controllo della Legionellosi* recommends a daily thermal shock at 65 °C for 30 min. In the Netherlands, for the installations where 60 °C cannot be achieved, a weekly thermal shock is required at different temperature–times (60 °C, 65 °C, 70 °C for 20, 10 and 5 min, respectively). In France, a generic daily thermal shock is also recommended for hot water systems (volume ≥ 400 L), where supply temperatures of ≥55 °C are not achieved. Therefore, many commercial accommodation sites have implemented their use. The industry, following these guides, is manufacturing and marketing water heaters that have integrated programs to automatically perform periodical thermal shocks, even for households. Interestingly, regulations and guidelines from other different countries do not include periodical thermal shock in their prevention strategies. This is the case for the United Kingdom [18,19] and Germany [20]. These dramatic differences between the different guidelines and regulations are striking and strongly suggest a lack of scientific consensus on this matter.

The basis for introducing this practice in guidelines and regulations is unclear, but the underlying theoretical idea is that regular pasteurization could prevent or reduce the colonization of the hot water system in a similar way to the thermal disinfection procedure. Thermal shock treatment at 70 °C to 80 °C for relatively short periods has been used for emergency disinfection for many years on the basis of the data showing logarithmic reduction kinetics of *Legionella pneumophila* sg1 as a function of increasing temperatures in laboratory conditions [21,22,23]. However, it should be noted that thermal shock, even when it is performed for disinfection purposes at full scale (>70 °C, extended recirculation periods and flushing of terminal points for a certain minimum time) has shown limited or insufficient efficacy [24,25]. In addition, in both pilot-scale studies and real-case scenarios, recolonization is common and can occur rapidly, even within days or a few weeks [26,27]. Different reasons have been adduced to explain this limited efficacy, including (a) structural deficiencies, e.g., dead legs and blind ends [28]; (b) *Legionella* survival in amoebal cysts [29]; (c) the presence of biofilms that may obstruct flow and heat transfer [30]; and (d) the persistence of viable but non-culturable (VBNC) *Legionella* cells resistant to high temperatures [31]. All this, combined with the technical difficulties of maintaining high temperatures throughout the treatment process, can contribute to the low efficacy of thermal disinfection procedures.

Moreover, in practice, both procedures (periodical thermal shock and thermal disinfection) are performed differently, so their results may differ. Most notably, during periodical thermal shocks, the terminal points are not flushed (they are usually conducted at night times when low use of hot water is expected), so only the hot water tanks and the recirculation line are thermally treated. In this way, the pipes that feed the end points (taps, showers) and the end points themselves are not treated during the process. Thus, it should be expected that in this part of the network, the colonization levels would not change significantly. Interestingly, in our study, in both groups A and B, the highest proportion of positive samples came from return lines (35.2% in group A and 44.4% in group B) and the lowest from calorifiers/hot water tanks (7.8% in group A and 12.0% in group B). This is probably because the samples from the calorifiers used to present the highest temperatures in the network, while those from the return line normally have the lowest temperatures, suggesting that the permanent maintenance of high temperatures could be more important for control than their periodical increase. In addition, the analysis of temperatures in the positive and negative samples reveals that the mean temperatures of the latter were, in both groups, higher than the former, which again highlights the role of temperature as a risk factor.

The results of our study suggest that the efficacy of regular thermal shocks, in terms of preventing *Legionella* colonization of the hot water systems in hotels, is limited. In effect, although the percentage of positive samples was lower in the group of hotels with thermal shock, the difference was not statistically significant. The same applies when considering only positive or negative sites, that is, hotels that show a certain degree of colonization (at least one sample positive). In short, regular thermal shock did not prevent presenting a significant proportion of positive samples (16.0% in the group with thermal shock) nor of having positive samples (32.5%). These results indicate per se that regular thermal shock was not effective for these purposes.

When our results were analyzed in terms of *Legionella* loads, the overall results did not show significant differences between the two groups. We also analyzed separately just the positive results (an endpoint not initially considered in the study). In this case, the *Legionella* positive results in the thermal shock group were significantly lower. However, this finding can be of low relevance: first, and most important, because this post hoc subgroup analysis does not alter the relevant conclusion derived from the whole group of quantitative results where no significant differences were found; and second, because only a very low proportion of the samples (2.1% in group A and 3.7% in group B) showed a concentration > 1000 UFC/L. This concentration, 1000 UFC/L, is the parametric value adopted by the current Directive (EU) 2020/2184 of the European Parliament and of the Council of 16 December 2020 on the quality of water intended for human consumption. We have to note that another study found higher loads in buildings where thermal shock was carried, although this study was done in single-family residences, and the mean of the hot water temperature was only around 50 °C versus around 55 °C in our study [15].

Our study has several strengths and some limitations. First, taking advantage of the fact that thermal shock in Spain is a “recommendation” and not a requirement, we had the opportunity to compare results from hotels with and without thermal shock. The average hot water temperatures and the temperature range at which the samples were taken in both groups did not differ significantly, thus allowing a comparative analysis between the two groups. It also benefits from the fact that all samplings and analysis were done by the same laboratory, thus avoiding the possible biases that could occur with different samplings and/or analysis systems in other studies. Finally, the methodology used for the thermal shock was essentially the same for all the sites, following the same guide (70 °C, 2 h recirculation).

The study also presents some limitations. First, both the number of hotels and the number of samples is too low for deriving clear conclusions. Further studies, with more hotels and more samples, would be necessary to obtain more statistically robust results. We also acknowledge that the frequency at which thermal shock was performed is heterogeneous, although in the majority (59.7%) of hotels, the procedure was carried out at monthly basis. Thus, regular thermal shocks could be more effective if it is performed more frequently, possibly daily or weekly. Finally, we cannot completely rule out some significant structural and operational differences between the two groups of hotels (size of the hotel, supplementary heating systems, piping materials, flushing regimes, chlorine levels, seasonality, etc.) that were not considered and could have conditioned the results in one way or another in both groups.

In summary, our study suggests that regular thermal shock has, at most, limited efficacy on the levels of *Legionella* colonization in hotels and does not support their use for long-term control purposes. Further investigations with more hotels and a larger number of samples are necessary to confirm these data. We note that eliminating unnecessary thermal shocks from guidelines and regulations would allow saving enormous energy costs and CO_2_ emissions and would avoid potential side effects, including risk of scalding, in the accommodation sector.

## 4. Materials and Methods

### 4.1. Experimental Design

The *Legionella* testing results from the period January–December 2019 coming from two groups of hotel facilities in the Balearic Islands (Spain) were analyzed. Group A included hotels that conducted regular thermal shocks along the period of study, and group B included hotels operating without regular thermal shocks. For group A, only hotels having a pertinent register of the performed thermal shocks were included. Differences between the two groups, in terms of both *Legionella* spp. positive results and positive sites were analyzed. *Legionella* samples were taken and tested by the same accredited laboratory (Biolinea Int. S.L., Palma, Spain).

### 4.2. Definitions

(a) Periodical thermal shock: thermal treatment of the domestic hot water system carried out at regular intervals (at least quarterly) by raising the temperature of the hot water storage heater at ≥70 °C, then keeping it in recirculation throughout the system for at least 2 h.
(b) Positive site: hotel with at least 1 positive sample for *Legionella* spp.

### 4.3. Sampling

The samplings were conducted in accordance with the Spanish standard UNE 100030:2017 (guidelines for prevention and control of proliferation and spread of Legionella in facilities). The number of samples collected from each hotel depended on the number of rooms being approximately 0.5$\;\sqrt{n}$ (n = number of rooms). All the samples were taken after 1 min of flushing, followed by measuring the temperature before collecting 1 L of water in a sterile container with sodium thiosulphate pentahydrate (0.01%, *w*/*v*) and transported immediately at ambient temperature into an isothermal bag to the laboratory.

### 4.4. Temperature Testing

Water temperature was tested using a calibrated digital thermometer at the time of the sample collection after 1 min of flushing.

### 4.5. Detection and Enumeration of Legionella spp. by Culture

The samples were processed within 24 h of collection. The detection and enumeration of *Legionella* spp. in the water samples was carried out according to the UNE-EN ISO 11731:2017 standard. Water samples where *Legionella* spp. was not detected or when containing < 10 CFU/L were considered negative, and the result equated to 0 CFU/L for the statistical analysis.

### 4.6. Statistical Analysis

For the statistical analysis, the 727 data were tested for normality using the Kolmogorov–Smirnov test. The nonparametric chi-squared test was used to compare the proportion of positive results and positive sites in the two groups. The Mann–Whitney U test was used to compare the loads of *Legionella* spp. Results with *p* < 0.05 were considered statistically significant.

## Figures and Tables

**Table 1:** Proportion of samples collected at the different temperature ranges in group A and group B.

Temperature Ranges (°C)	Group A (%)	Group B (%)
<50	8.1	10.1
50–54	27.2	32.6
55–60	43.3	47.2
>60	21.3	10.1

**Table 2:** Proportion of positive samples in groups A and B in relation to the main sampling points. N = Number of samples for each sampling point; n = number of positives; (%) = proportion of positive samples for each sampling point.

	Group A			Group B		
	Samples	Positive		Samples	Positive	
	N	n	(%)	N	n	(%)
**Showers**	181	36	(19.8)	142	23	(16.1)
**Taps**	116	18	(15.5)	80	25	(31.2)
**Calorifiers/hot water tanks**	115	9	(7.8)	58	7	(12.0)
**Return line**	17	6	(35.2)	18	8	(44.4)

**Table 3:** Average temperatures of *Legionella* spp. positive and negative samples of groups A and B.

	Group A		Group B	
	Positive Samples	Negative Samples	Positive Samples	Negative Samples
**Temperature (°C)**	52.9 ± 6.6	56.3 ± 5.9	53.1 ± 3.6	55.8 ± 5.9

**Table 4:** Summary of *Legionella* spp. concentrations results in groups A and B. n = number of samples within each count range; % = proportion of results in each count range referred to the total number of samples of the group.

*Legionella* spp. (CFU/L)	Group A			Group B		
	n	%	Mean (CFU/L)	n	%	Mean (CFU/L)
≤1 × 10^2^	45	10.5	21 ± 16	30	10.1	27 ± 23
1 × 10^2^–1 × 10^3^	15	3.5	1.9 × 10^2^ ± 1.1 × 10^2^	22	7.4	1.8 × 10^2^ ± 91
≥1 × 10^3^	9	2.1	3.3 × 10^4^ ± 4.8 × 10^4^	11	3.7	4.3 × 10^4^ ± 5.0 × 10^4^
Negative	360	83.9	-	235	78.8	-

## Data Availability

Not applicable.
